# Coencapsulation of Polyphenols and Anthocyanins from Blueberry Pomace by Double Emulsion Stabilized by Whey Proteins: Effect of Homogenization Parameters

**DOI:** 10.3390/molecules23102525

**Published:** 2018-10-02

**Authors:** Bio Sigui Bruno Bamba, John Shi, Carole C. Tranchant, Sophia Jun Xue, Charles F. Forney, Loong-Tak Lim, Weili Xu, Guihua Xu

**Affiliations:** 1 Department of Biochemistry and Genetics, Biological Sciences Training and Research Unit, Université Peleforo Gon Coulibaly, Korhogo BP 1328, Côte d’Ivoire; 2 Agriculture and Agri-Food Canada, Guelph Research and Development Centre, Guelph, ON N1G 5C9, Canada; jun.xue@agr.gc.ca (S.J.X.); weili.xu@agr.gc.ca (W.X.); guihua.xu@agr.gc.ca (G.X.); 3 School of Food Science, Nutrition and Family Studies, Université de Moncton, Moncton, NB E1A 3E9, Canada; 4 Agriculture and Agri-Food Canada, Kentville Research and Development Centre, Kentville, NS B4N 1J5, Canada; charles.forney@agr.gc.ca; 5 Food Science Department, University of Guelph, Guelph, ON N1G 2W1, Canada; llim@uoguelph.ca

**Keywords:** double emulsion, nanoencapsulation, blueberry pomace extract, phenolic compounds, anthocyanins, homogenization conditions, microfluidization, whey proteins, polyglycerol polyricinoleate

## Abstract

Blueberry pomace is a rich source of high-value bioactive polyphenols with presumed health benefits. Their incorporation into functional foods and health-related products benefits from coencapsulation and protection of polyphenol-rich extracts in suitable carriers. This study aimed to create a water-in-oil-in-water (W_1_/O/W_2_) double emulsion system suitable for the coencapsulation of total phenolics (TP) and anthocyanins (TA) from a polyphenol-rich extract of blueberry pomace (W_1_). The effect of critical physical parameters for preparing stable double emulsions, namely homogenization pressure, stirring speed and time, was investigated by measuring the hydrodynamic diameter, size dispersity and zeta potential of the oil droplets, and the encapsulation efficiency of TP and TA. The oil droplets were negatively charged (negative zeta potential values), which was related to the pH and composition of W_2_ (whey protein isolate solution) and suggests stabilization by the charged whey proteins. Increasing W_1_/O/W_2_ microfluidization pressure from 50 to 200 MPa or homogenization speed from 6000 to 12,000 rpm significantly increased droplet diameter and zeta potential and decreased TA and TP encapsulation efficiency. Increasing W_1_/O/W_2_ homogenization time from 15 to 20 min also increased droplet diameter and zeta potential and lowered TA encapsulation efficiency, while TP encapsulation did not vary significantly. In contrast, increasing W_1_/O homogenization time from 5 to 10 min at 10,000 rpm markedly increased TA encapsulation efficiency and reduced droplet diameter and zeta potential. High coencapsulation rates of blueberry polyphenols and anthocyanins around 80% or greater were achieved when the oil droplets were relatively small (mean diameter < 400 nm), with low dispersity (<0.25) and a high negative surface charge (−40 mV or less). These characteristics were obtained by homogenizing for 10 min at 10,000 rpm (W_1_/O), then 6000 rpm for 15 min, followed by microfluidization at 50 MPa.

## 1. Introduction

Polyphenols are known for their strong antioxidant properties and potential health benefits, including the prevention of diet-related chronic illnesses such as type 2 diabetes, cardiovascular diseases, neurodegenerative diseases and some cancers [1]. Polyphenols encompass a very diverse group of molecules, which include phenolic acids, flavonoids (e.g., anthocyanidins and anthocyanins) and many others. Since they are produced by plants, they can be obtained through the diet by regular consumption of plant-based foods, specifically fruits, vegetables, cereal grains and certain beverages (e.g., tea and wine) [1]. However, the contents and types of phenolic compounds in these foods vary greatly [1,2], which can limit their dietary intake. Increasingly, the food and pharmaceutical industries aim to deliver these health-promoting compounds to consumers in various forms, such as fortified foods, functional foods and beverages as well as dietary supplements. This presents considerable challenges, however, as polyphenols are generally unstable following their extraction from plant materials. They are prone to undesired inactivation or degradation under conditions that typically occur in foods and during food processing, such as low or high pH, heat, presence of enzymes, proteins, metallic ions, oxygen and light [3], leading to losses in their biological activity and functionality. In addition, many phenolic compounds have a relatively low solubility in food matrices and low bioavailability in humans [1,2], which limits their potential health benefits. Some have an unpleasant odour, bitter taste and astringency [4,5].

Microencapsulation has proven an excellent method to protect food ingredients against adverse reactions, deterioration and undesirable interactions with other ingredients, to improve their solubility [6,7] and mask unpleasant off-flavours [5]. The latest evidence on the encapsulation of bioactive molecules suggests that coencapsulation (i.e., the simultaneous encapsulation of several compounds in a single encapsulation carrier) can improve the bioactivity of the individual compounds due to synergistic effects [8]. Polyphenol-rich extracts typically contain various phenolic compounds, which may promote the health benefits of the extracts due to synergistic action among their constituents [2,9,10]. In animal models and preliminary human trials, phenolic-rich products from blueberry (*Vaccinium* section *Cyanococcus* spp.) were found to be more effective than individual purified phenolics (e.g., anthocyanins) in altering the development of obesity, type 2 diabetes, chronic inflammation and health-related metabolic factors [11,12,13].

Multicore microencapsulation is a promising method for the simultaneous encapsulation of bioactive compounds. Among the various microencapsulation technologies that have been developed, emulsion-based encapsulation is considered as one of the most promising for the protection and delivery of polyphenols because relatively high encapsulation efficiency, stability and effective controlled release can be achieved under certain conditions [14]. This technique relies on the formation of simple emulsions, multiple (double) emulsions, or nanoemulsions. It involves relatively simple processing and low energy costs, and benefits from the fact that emulsions (conventional simple emulsions mainly) have been used in food, pharmaceutical and cosmetic applications for many years [15]. Double emulsions such as water-in-oil-in-water (W_1_/O/W_2_) emulsions are multi-compartmentalized (multicore) systems consisting of a water-in-oil (W_1_/O) emulsion dispersed as droplets in a continuous aqueous phase (W_2_) [16]. Double emulsion processing can give quite stable, well-defined systems with reproducible particle sizes [17]. However, they remain prone to the same types of physical instability as conventional emulsions (creaming, flocculation, coalescence and Ostwald ripening, which can affect both the oil and water droplets), as well as additional instability mechanisms associated with the presence of the inner water phase (i.e., diffusion of water between the inner and outer aqueous phases and expulsion of water droplets from the oil droplets) [16].

Physical instability is inherent to emulsion systems but it can be delayed by several parameters, mainly emulsion composition and homogenization conditions [16,18,19]. Nanoemulsions refer to emulsion systems (simple or multiple) in which the dispersed droplets range from 50 to 200 nm in diameter (transparent emulsions) or up to 500 nm resulting in a milky appearance [20]. Nanoscale droplets can be obtained by high-flow homogenization (e.g., high-pressure homogenization). Nanoemulsions are more stable to gravitational separation (creaming) and flocculation than conventional emulsions because of the relatively small droplet size [14]. They also necessitate lower amounts of emulsifiers [21]. Most studies on food-grade double emulsions have focused on emulsion composition [18,19,22,23,24], while fewer have examined the effect of physical processing parameters [15,25,26,27,28,29]. Other studies were conducted with organic solvents [30,31]. They suggest that the effects of homogenization speed and time, along with pressure when applicable, are greatly influenced by the emulsion system and process considered, which vary widely among studies. There is a need to better understand the influence of processing conditions on the characteristics and behaviour of food-grade double emulsions in relation to the encapsulation of bioactive molecules. To the best of our knowledge, no study has investigated the effect of homogenization parameters on the coencapsulation of phenolic compounds from blueberry by double emulsion.

The present study aimed to create a W_1_/O/W_2_ double nanoemulsion system suitable for the coencapsulation of total phenolics (TP) and total anthocyanins (TA) extracted from blueberry pomace. The specific objective was to assess the effects of homogenization pressure, stirring speed and time on the characteristics of the double emulsion, namely particle size, size dispersity and zeta potential of the oil droplets, and efficiency of TP and TA encapsulation. These characteristics greatly impact the stability and functional performance of nanoemulsions (e.g., appearance, texture and bioavailability) and of the final products in which they are used. The double emulsion contained a polyphenol-rich aqueous extract of blueberry pomace (W_1_), which was emulsified in corn oil with polyglycerol polyricinoleate (PGPR) to form the primary emulsion W_1_/O, which was subsequently dispersed in a whey protein isolate (WPI) solution (W_2_) to form the double emulsion. In keeping with the green extraction technology which was developed to extract the phenolic compounds from blueberry pomace [32], the other ingredients of the emulsions were selected because they are safe for human consumption and environmentally friendly. The emulsifiers PGPR and WPI are both generally recognized as safe (GRAS denomination) [33,34]. WPI is further recognized as an environmentally friendly stabilizer (green biopolymer) [19]. Likewise, the use of blueberry pomace is a strategy that aims to valorise this polyphenol-rich by-product.

## 2. Results and Discussion

This study is the first to examine the influence of homogenization conditions on the coencapsulation of total phenolics and anthocyanins from blueberry pomace by double emulsion. The homogenization parameters that were varied are the stirring speed and time used to prepare the coarse W_1_/O/W_2_ double emulsion, the microfluidization pressure used to prepare the final double emulsion, and the stirring time used to prepare the primary W_1_/O emulsion, as summarized in Table 1. Four series of experiments were performed to identify suitable levels for each parameter.

### 2.1. Effect of W_1_/O/W_2_ Homogenization Pressure on the Characteristics of the Double Emulsion

**Figure 1:**
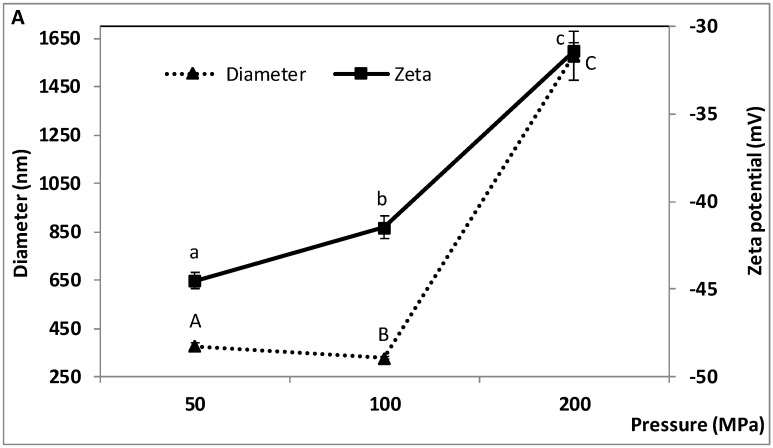

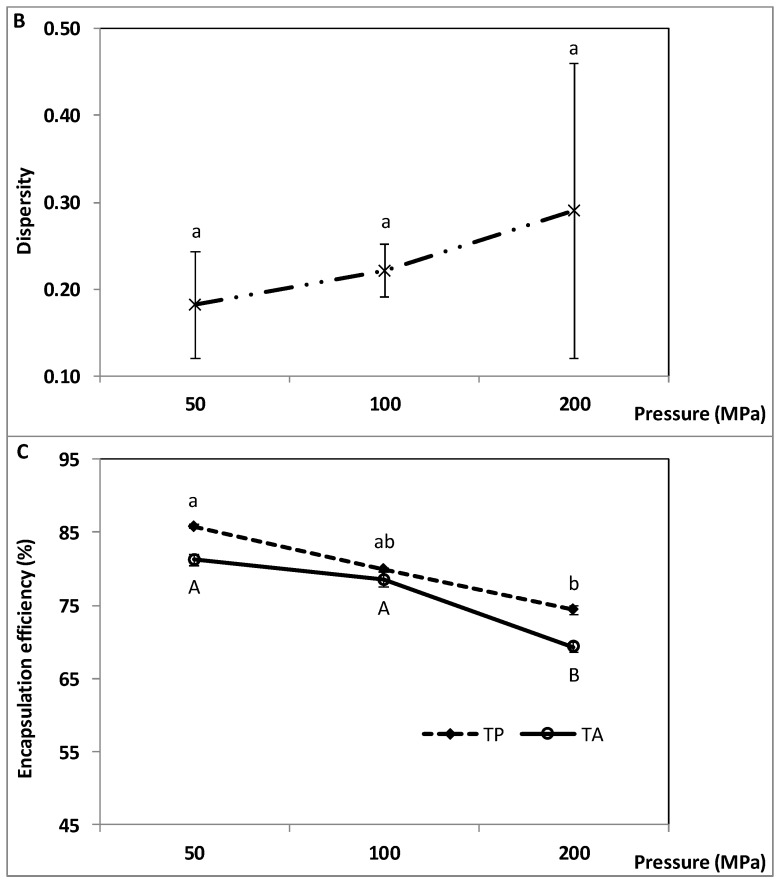
Effect of W_1_/O/W_2_ homogenization pressure on the characteristics of the double emulsion, (**A**) droplet diameter, zeta potential, (**B**) size dispersity, and (**C**) encapsulation efficiency of total polyphenols (TP) and total anthocyanins (TA). W_1_/O homogenization speed and time: 10,000 rpm and 10 min; W_1_/O/W_2_ homogenization speed and time: 6000 rpm and 15 min. Means (three replicates) ± standard deviation (SD). Means with different letters in each series are significantly different (*p* ≤ 0.05).

The effect of high-pressure homogenization was assessed by varying the microfluidizer pressure from 50 to 200 MPa with one cycle through the microfluidizer after homogenization at 10,000 rpm for 10 min (W_1_/O) and 6000 rpm for 15 min (W_1_/O/W_2_). During microfluidization, the high energy input forces the droplets in the pre-emulsion through a small diameter die. The total force applied to the system, which depends on the microfluidization pressure and number of cycles through the microfluidizer, influences the final droplet size. As illustrated in Figure 1, the diameter of the oil droplets, their zeta potential and the encapsulation efficiency of TP and TA were significantly affected by microfluidization pressure, while the effect on size dispersity was not statistically significant. Droplet diameter decreased from 377 ± 11.5 to 327 ± 5.46 nm between 50 and 100 MPa, then increased to 1580 ± 100 nm when the pressure was raised to 200 MPa (Figure 1A). Size dispersity, which ranged from 0.18 to 0.29 between 50 and 200 MPa, exhibited a high variability at 200 MPa, as indicated by the elevated standard deviation (SD) (Figure 1B). The zeta potential, i.e., the net surface charge of the oil droplets, increased from −44.53 ± 0.47 to −31.43 ± 0.50 mV between 50 and 200 MPa (Figure 1A). The zeta potential of the bulk WPI solution (pH 6.8) used to prepare the emulsions was −17.37 ± 0.64 mV. The encapsulation efficiency of TP and TA was significantly higher at 50 MPa (85.7 ± 0.29% and 81.2 ± 0.73%, respectively) than at 200 MPa (74.3 ± 0.61 and 69.2 ± 0.75%, respectively) (Figure 1C).

The results show that double nanoemulsions with relatively small particle size (average diameter less than 400 nm), relatively low size dispersity (below 0.25) and high negative surface charge (below −40 mV) were obtained at 50 and 100 MPa, suggesting that microfluidization under these conditions was effective at producing fine emulsions by breaking down the oil droplets to the nanoscale. The interfacial tension between the oil and water phases may have been lower at 50 and 100 MPa than at 200 MPa, due to greater emulsifying capacity of the whey proteins (WPI) at 50–100 MPa, thus facilitating the production of small droplets by lowering the cohesive or restorative forces (determined by the Laplace pressure) that oppose the breakage of the droplets [35]. The milky appearance of all the nanoemulsions throughout our study is consistent with the values of average particle diameter. The net negative charge of the oil droplets can be explained by the adsorption of negatively charged whey proteins on the surface of the droplets, which would contribute to their stabilization by electrostatic repulsion and steric hindrance. Under the near neutral pH conditions used in our study, WPI carries a negative net charge due to its negatively charged carboxyl groups at pH above 5 [36]. At room temperature, the hydrophobic interactions that enable the adsorption of whey proteins at the oil-water interface are relatively strong [37].

The marked increase in droplet diameter and the greater variability (SD) of particle size and dispersity at 200 MPa are suggestive of overprocessing, a phenomenon whereby the average droplet size increases with homogenization pressure [21,38]. This indicates that the rate of droplet coalescence becomes greater than the rate of droplet breakage. For simplification, the term coalescence is used to refer to two distinct processes (coalescence and Ostwald ripening) as they both lead to an increase in particle size but cannot be easily distinguished [39]. Overprocessing can be attributed to the limited ability of the emulsifier to rapidly adsorb onto the newly created surfaces and to the high Brownian motion, which increase the probability of collisions leading to droplet coalescence at high microfluidization pressure [40]. When the timescale of collisions is shorter than that of adsorption, the interfaces of the newly formed oil droplets cannot be fully covered by the emulsifier (WPI in our study), resulting in insufficient droplet stabilization and increased coalescence [21,40]. Denaturation of the whey proteins may be a contributing factor, further decreasing the ability of WPI to effectively stabilize the droplets at 200 MPa. Denaturation of WPI may result from the force-induced phenomena of cavitation, shear and turbulence and from the temperature rise that occur simultaneously in the homogenization chamber during microfluidization [41,42]. Although the microfluidizer enables sample temperature to be regulated during processing, temperature increase at high pressure cannot be ruled out. Reduced emulsifying efficacy of WPI at 200 MPa would be consistent with the marked increase in zeta potential (from −44 to −31 mV) evidenced at 200 MPa. Less negative zeta potential may also be related to smaller total surface area (higher droplet size). A less net negative surface charge would reduce the electrostatic repulsion, thus promoting droplet coalescence. Lower viscosity of the continuous water phase (WPI solution), such as may occur if the temperature increases or upon WPI denaturation in the homogenization chamber, or when particle size increases [43], would also promote coalescence. Lower viscosity is also expected to make it more difficult to break down the droplets in the first place [35].

To date, overprocessing during high-pressure emulsification has been reported mostly in simple emulsions, mainly O/W [20,21,38] and more rarely W/O [40]. The pressure ranges at which particle size was found to increase in these studies vary from 63–84 MPa [21], 69–105 MPa [37], 70–105 MPa [20], 90–150 MPa [38] and 150–300 MPa [41]. This suggests that the pressure at which overprocessing may occur depends on the emulsion system considered, including homogenization conditions, equipment and emulsion composition. In our study, the value of pressure at which overprocessing began (between 100 and 200 MPa) cannot be identified precisely as we only tested three levels of pressure. Further study would be needed to find out. Characterization of particle size distribution would also provide additional insights. The smallest values of average diameter reported in previous studies, which generally ranged from 200–550 nm for simple emulsions [20,21,38,40,41], are comparable to the values obtained in the present work with double emulsions.

It is noteworthy that the experimental conditions used in previous studies are quite different. Simple O/W emulsions have been studied the most. Other differences include pressure range, type of microfluidizer, number of homogenization cycles and emulsion composition. In some studies, no detrimental effect of high-pressure homogenization (up to 150 MPa or greater) on particle size was found [44,45,46,47]. Wang et al. [25] found no adverse effect of homogenization pressure on oil droplet size in W_1_/O/W_2_ double emulsions in the range 5 to 65 MPa (first emulsification step, four passes) and 5 to 20 MPa (second step, three passes). They suggested that the double emulsions were stabilized when the magnitude of the zeta potential exceeded 30 mV and reported values around −40 mV with WPI. These values are consistent with ours in the range 50 to 100 MPa. However, the lowest particle diameters in their work (1605 nm when PGPR and WPI were used as emulsifiers and above 850 nm when PGPR and Tween 80 were used) [25] were notably higher than in the present study.

At 200 MPa, the reduction in TP and TA encapsulation efficiency evidenced in our work may be due in part to the altered size and charge characteristics of the oil droplets, possibly related to a reduction of WPI emulsifying capacity. Droplet coalescence may be conducive to some losses of encapsulated compounds. This may occur to a greater extent if the inner water droplets (W_1_/O emulsion) were partially destabilized, damaged or expelled from the oil droplets when subjected to the highest microfluidization pressure, due to high shear and temperature rise, thus leading to the loss of some encapsulated TP and TA during the second homogenisation step. The bioactivity of the phenolic compounds may also be affected, which could influence their quantification, with possible underestimation of their concentration. However, these losses seemed relatively small as encapsulation efficiencies above 65% were retained at 200 MPa. TP and TA are supposed to be encapsulated mainly in the inner aqueous phase (water droplets) but it cannot be ruled out that some may have diffused to the oil droplets, then possibly to the outer aqueous phase. Their bioactivity upon encapsulation and their distribution between the different phases warrant further investigation.

The relatively high encapsulation efficiencies in our work (69–85%) compare favorably with previous reports. To our knowledge, there is no study of the effect of homogenization pressure on polyphenol coencapsulation by double emulsion. Wang et al. [25] reported an efficiency of 94.9% when encapsulating a pure phenolic compound (*trans*-resveratrol) in a W_1_/O/W_2_ double emulsion. The homogenization conditions and emulsion composition were different than in the present work and resulted in large oil droplets (>850 nm) [25]. Akhtar et al. [48] reported efficiencies of 72 and 80%, respectively, when encapsulating anthocyanin-rich Rosella extracts or rutin in double emulsions prepared using a spinning disc reactor in the second emulsification step. With green tea polyphenols and different encapsulation methods (i.e., freeze drying and spray drying), encapsulation efficiencies of 60–76% and 40–70%, respectively, were found by Pasrija et al. [49]. da Rosa et al. [50] encapsulated blackberry phenolic compounds by freeze drying and reported important variations in encapsulation efficiencies depending in part on the phenolic compound (from 0–14% for *p*-hydroxybenzoic acid, 24–51% for epicatechin and 46–75% for gallic acid, depending on the encapsulation matrix).

In the present study, double nanoemulsions with relatively small particle size, low size dispersity, high negative surface charge, and encapsulation efficiency of TP and TA around 80% or greater were consistently obtained at 50 and 100 MPa. Slightly finer emulsions were obtained at 100 MPa, but 50 MPa was found to be a good compromise as it resulted in high encapsulation efficiencies similar to those obtained at 100 MPa, while lowering the energy consumption of the homogenization process by operating at a lower pressure. A pressure of 50 MPa was used in subsequent experiments.

### 2.2. Effect of W_1_/O/W_2_ Homogenization Stirring Speed on the Characteristics of the Double Emulsion

**Figure 2:**
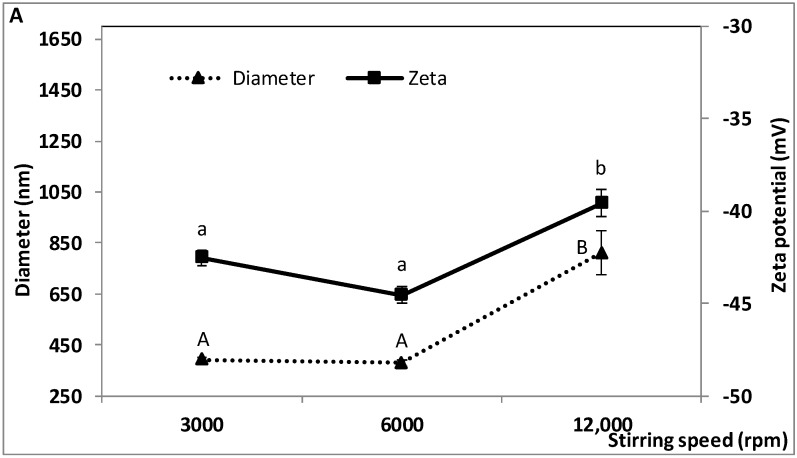

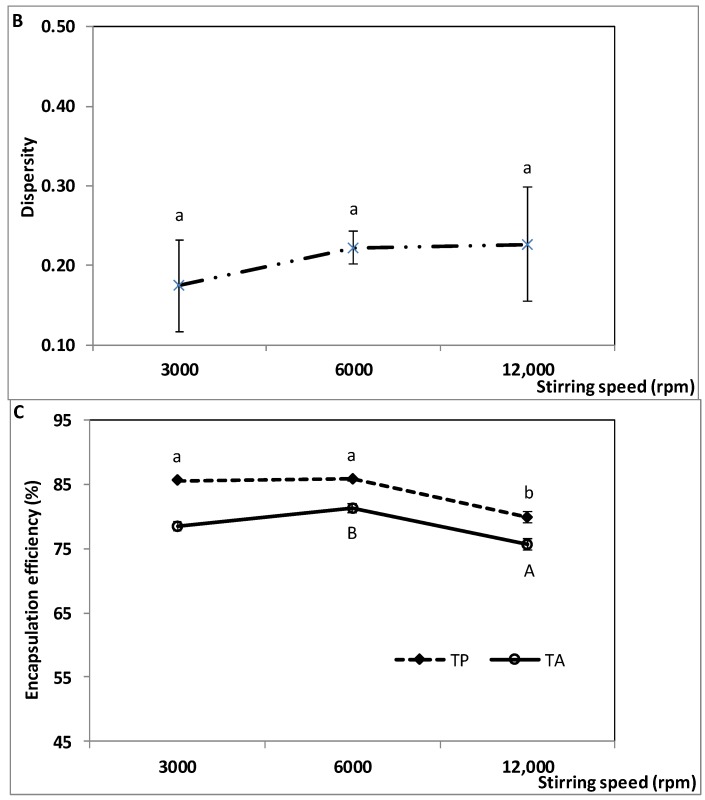
Effect of W_1_/O/W_2_ homogenization stirring speed on the characteristics of the double emulsion, (**A**) droplet diameter, zeta potential, (**B**) size dispersity, and (**C**) encapsulation efficiency of total polyphenols (TP) and total anthocyanins (TA). W_1_/O homogenization speed and time: 10,000 rpm and 10 min; W_1_/O/W_2_ homogenization time and microfluidization pressure: 15 min and 50 MPa. Means (three replicates) ± SD. Means with different letters in each series are significantly different (*p* ≤ 0.05).

The effect of stirring speed during the second homogenization step before microfluidization was investigated in the range 3000 to 12,000 rpm. Mixing time (15 min) and all the other parameters were kept constant. As shown in Figure 2, the size and zeta potential of the oil droplets and the encapsulation efficiency of TP and TA were significantly affected by stirring speed. The effect on size dispersity was not statistically significant. Droplet size and zeta potential did not vary significantly between 3000 and 6000 rpm but significantly increased at 12,000 rpm (Figure 2A). The corresponding values were 390 ± 11.8, 377 ± 11.5 and 809 ± 86.4 nm for diameter and −42.53 ± 0.42, −44.53 ± 0.47 and −39.57 ± 0.75 mV for zeta potential. Size dispersity ranged from 0.17 ± 0.06 to 0.23 ± 0.07 between 3000 and 12,000 rpm (Figure 2B). The highest values of encapsulation efficiency were obtained at 3000 and 6000 rpm for TP (85.5 ± 0.20 and 85.7 ± 0.29%) and at 6000 rpm for TA (81.2 ± 0.73%) (Figure 2C).

These findings show that well-controlled double emulsions were obtained at 3000 and 6000 rpm, suggesting that both stirring speeds imparted enough mechanical energy to break down the oil droplets and produce small droplets (<400 nm). Thus, the disruptive forces generated by mixing at both speeds probably exceeded the cohesive forces. When stirring is energetic enough, the droplet and eddy scale is reduced, and translational Brownian motion of the emulsifier is high because surfactant diffusion from the bulk is partly substituted by convection, which enables rapid adsorption of the emulsifier at the oil-water interface [51]. High stirring speeds, however, also increase the likelihood of droplet coalescence by increasing droplet velocity and collision rate. Therefore, an appropriate balance must be achieved so that the droplet size remains small.

Increased particle size at 12,000 rpm suggests that coalescence was promoted at the expense of particle break down. Droplet coalescence may have been promoted by the temperature rise during mixing. At 12,000 rpm, the emulsion flask warmed up noticeably during mixing. High shear and rising temperature at this speed may have induced some denaturation of WPI, decreasing its ability to effectively stabilize the newly created oil droplets. Lower viscosity of the continuous aqueous phase (WPI solution), such as may occur with rising temperature or upon WPI denaturation, would also promote droplet coalescence. As reported by Wang et al. [52] and Pal [53], double emulsions are non-Newtonian fluids and behave as more dilute under high shear, which is similar to shear thinning. Lower viscosity of the WPI solution would also make it more difficult to break down the droplets [35]. Reduced emulsifying efficacy of WPI would be consistent with the less negative zeta potential evidenced at 12,000 rpm, which would promote coalescence by reducing electrostatic stabilization.

Our findings indicate that increasing the homogenization speed is beneficial only when a critical speed is not exceeded, in agreement with previous findings regarding particle size and emulsion stability. Most studies have focused on simple emulsions with great variations in stirring speeds (e.g., 100–1300 rpm [51] and 10,000–15,000 rpm [54]) and emulsion composition. Liyana et al. [54] found that the stability of O/W emulsions was improved by increasing the stirring speed from 10,000 to 15,000 rpm, but they did not measure particle size. With O/W Pickering emulsions, Tsabet and Fradette [55] found that increasing the stirring speed increased the effectively covered interface, leading to more stable emulsions. However, beyond a certain speed, the effectively covered interface decreased. With a W_1_/O/W_2_ double emulsion, Ahmed et al. [31] reported a decrease in particle size when stirring speed increased in the range 10,000–20,000 rpm, followed by an increase in size at 24,000 rpm.

At 12,000 rpm, the reduction in TP and TA encapsulation efficiency may be explained in part by the altered size and charge characteristics of the oil droplets. Droplet coalescence and modification of the bioactivity of phenolic compounds, which could lead to some losses of encapsulated phenolics and anthocyanins as discussed in Section 2.1, may occur to a greater extent upon mixing at this high speed due to high shear and rising temperature. These losses seemed small, however, as encapsulation efficiencies of 75% or greater were obtained at 12,000 rpm. The relatively high efficiency of encapsulation in the present study (75–85%) is comparable to or higher than in previous reports, which ranged from 0–75% [50], 40–76% [49], 72–80% [48] to 94.9% [25] depending on the encapsulation method and nature of the compound(s) that were encapsulated. None of these studies assessed the effect of homogenization speed on encapsulation yield by double emulsion. In the present work, a speed of 6000 rpm during the second homogenization step was found to be a suitable compromise, resulting in average particle size below 400 nm and encapsulation efficiencies around 80% or greater. This speed was used in subsequent experiments.

### 2.3. Effect of W_1_/O/W_2_ Homogenization Stirring Time on the Characteristics of the Double Emulsion

**Figure 3:**
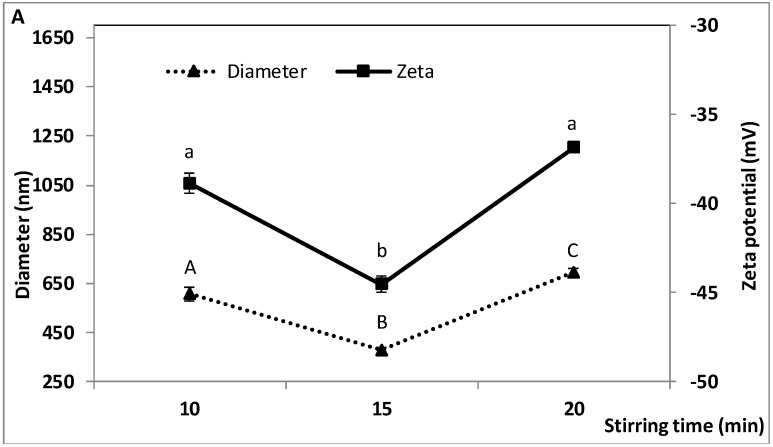

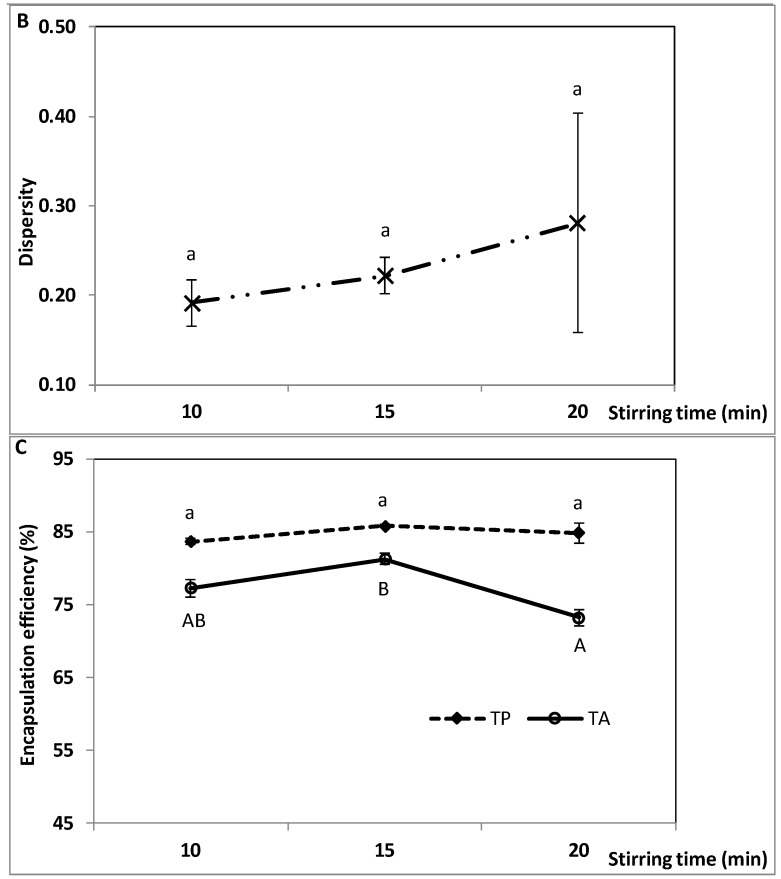
Effect of W_1_/O/W_2_ homogenization stirring time on the characteristics of the double emulsion, (**A**) droplet diameter, zeta potential, (**B**) size dispersity, and (**C**) encapsulation efficiency of total polyphenols (TP) and total anthocyanins (TA). W_1_/O homogenization speed and time: 10,000 rpm and 10 min; W_1_/O/W_2_ homogenization speed and microfluidization pressure: 6000 rpm and 50 MPa. Means (three replicates) ± SD. Means with different letters in each series are significantly different (*p* ≤ 0.05).

Stirring time during the second homogenization step was varied from 10 to 20 min, while stirring speed (6000 rpm) and all the other parameters were kept constant. As shown in Figure 3, droplet diameter, zeta potential and TA encapsulation efficiency were significantly affected by stirring time. In contrast, size dispersity and TP encapsulation efficiency did not vary significantly. Particle diameter and zeta potential exhibited a V-shaped profile with minimum values (377 ± 11.5 and −44.53 ± 0.47, respectively) when homogenizing for 15 min (Figure 3A). They varied from 607 ± 29.0 to 693 ± 15.8 nm and −38.87 ± 0.55 to −36.87 ± 0.15, respectively, between 10 and 20 min. Size dispersity ranged from 0.19 ± 0.03 to 0.28 ± 0.12 (Figure 3B). TP encapsulation efficiency remained unchanged, ranging from 83.6 ± 0.38 to 85.7 ± 0.29%, while TA encapsulation was greater after 15 min (81.2 ± 0.73%) than after 20 min (73.2 ± 1.02%) (Figure 3C).

The results show that increasing stirring time is beneficial for producing fine double emulsions only when a critical duration is not exceeded. This is consistent with previous findings, but most were obtained with simple emulsions. With W/O Pickering emulsions prepared at 10,300 rpm, Sawiak et al. [56] observed a decrease in droplet radius between 1 and 15 min, followed by an increase between 15 and 25 min. A similar trend was reported by Ahmed et al. [31] with a double emulsion, as particle size began to increase when stirring time exceeded 15 min. Increasing mixing time supplies more energy to break down the droplets, resulting in smaller droplets, but beyond a certain time, Sawiak et al. [56] argued that larger droplets begin to form due to the exhaustion of surfactant particles. In our study, the increase in droplet size and the greater variability (SD) of size dispersity evidenced between 15 and 20 min suggests a decreased ability of WPI to stabilize the newly formed oil droplets. The explanation for the adverse effect of prolonged mixing on droplet size and charge may be similar to the explanation we offered to explain the detrimental effects of high homogenization speed and high microfluidization pressure. Briefly, the combined effects of high shear, temperature rise and decreased viscosity of the continuous aqueous phase may favour droplet coalescence at the expense of small droplet production.

High TP encapsulation efficiency (around 85%) is probably due in part to the characteristics of the droplets, which enable a high retention of total phenolics in the inner aqueous phase. Unlike TP, TA encapsulation efficiency was slightly reduced at a stirring time of 20 min that resulted in larger droplets, suggesting that anthocyanins may be more sensitive to the effect of mixing time (and droplet characteristics) than TP. The fact that anthocyanins are generally more water soluble than other flavonoids and phenolics [48] may facilitate their diffusion from the inner water droplets (W_1_/O) to the outer aqueous phase of the double emulsion when this emulsion is made, as the vigorous mixing required to prepare the double emulsion may partly damage or destabilize the PGPR-stabilized water droplets, or expel some of them from the oil droplets, leading to some loss of encapsulated compounds. The possible differential behaviour of TA and TP in terms of their encapsulation and release by double emulsion is intriguing and warrants further investigation. Important variations in the encapsulation efficiency of blackberry phenolics by freeze drying, which depended in part on the phenolic compound, have been reported by da Rosa et al. [50]. In the present work, encapsulation efficiencies around 80% or greater for both TP and TA were obtained when mixing time was limited to 15 min during the second homogenization step, which produced fine double emulsions (average particle size < 400 nm and dispersity < 0.25).

### 2.4. Effect of W_1_/O Homogenization Stirring Time on the Characteristics of the Double Emulsion

**Figure 4:**
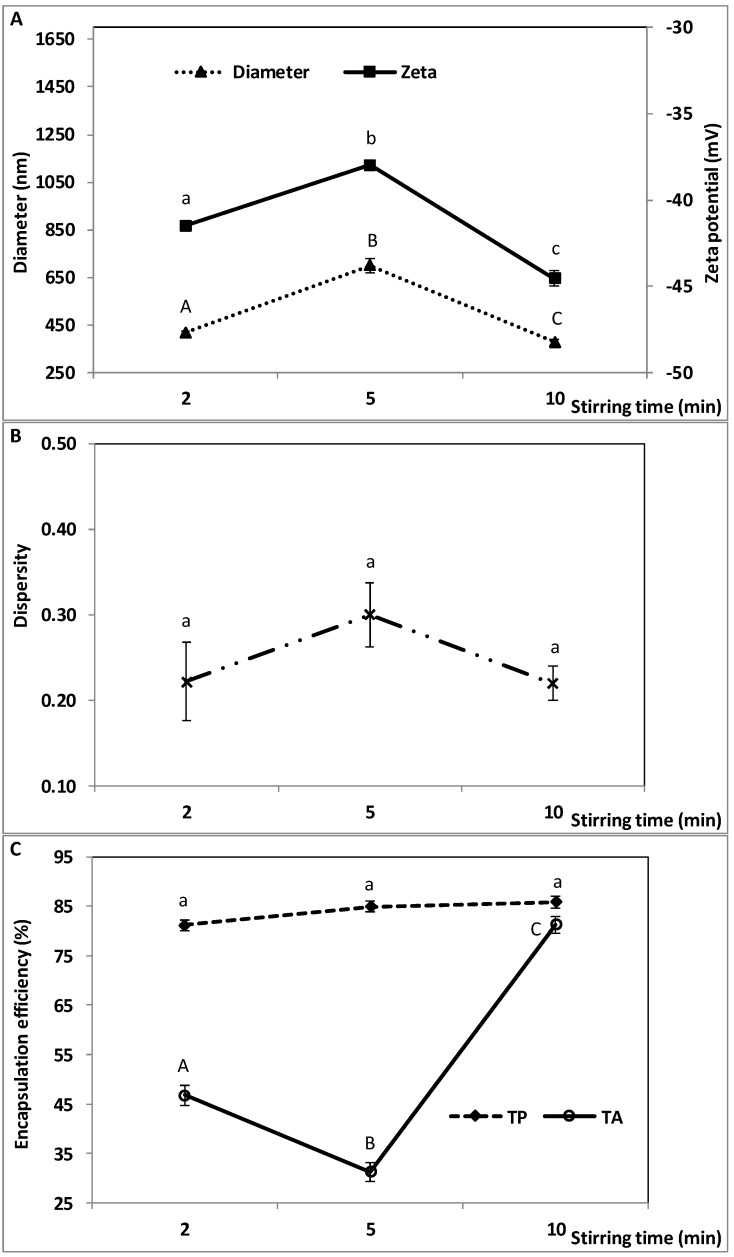
Effect of W_1_/O homogenization stirring time on the characteristics of the double emulsion, (**A**) droplet diameter, zeta potential, (**B**) size dispersity, and (**C**) encapsulation efficiency of total polyphenols (TP) and total anthocyanins (TA). W_1_/O homogenization speed: 10,000 rpm; W_1_/O/W_2_ homogenization speed, time and microfluidization pressure: 6000 rpm, 15 min and 50 MPa. Means (three replicates) ± SD. Means with different letters in each series are significantly different (*p* ≤ 0.05).

Stirring time during homogenization of the oil phase and the W_1_ phase at 10,000 rpm was varied in the range 2 to 10 min. An extra parameter was varied during the premixing step of oil and emulsifier (PGPR). Prior to homogenization for 2 min, PGPR was mixed with oil at 10,000 rpm for 2 min. Prior to homogenization for 5 or 10 min, PGPR and oil were mixed at 5000 rpm for 5 min. This difference may influence the characteristics of the final double emulsion. The other parameters were kept constant. The oil droplet diameter, zeta potential and TA encapsulation efficiency varied significantly with W_1_/O stirring time, while size dispersity and TP encapsulation efficiency did not (Figure 4). Particle size and zeta potential were the lowest at 2 and 10 min (418 ± 5.19 and 377 ± 11.5 nm and −41.50 ± 0.21 and −44.53 ± 0.47 mV, respectively). TA encapsulation efficiency decreased (46.8 to 31.2%) between 2 and 5 min, then increased to 81.2% at 10 min. TP encapsulation was fairly constant (81.1–85.7%).

These findings indicate that W_1_/O homogenization time must be long enough (10 min in the present study) to form double emulsions with small particle size (<400 nm on average), small dispersity (<0.25) and highly negative surface charge (below –40 mV). A suitable homogenization time with vigorous stirring would enable the emulsifier (PGPR) to effectively cover the water-oil interface of the newly formed water droplets. As droplet size decreases, the viscosity of the emulsion may increase slightly, as reported by Pal [43], further stabilizing the primary emulsion by hindering coalescence. Effective stabilization of the water droplets in the W_1_/O emulsion may enable effective encapsulation of the phenolic compounds and promote the formation of fine and well-controlled double emulsions. This may explain the differences found between 5 and 10 min in terms of the characteristics of the double emulsions.

At the lowest W_1_/O homogenization time of 2 min, PGPR would have had less time to cover and stabilize the interface. However, because the premixing of PGPR and oil was performed at a higher speed (10,000 rpm), it can be surmised that the emulsifier was well dispersed in the oil before making the primary emulsion. This may have enhanced PGPR efficacy to stabilize the water droplets, even though W_1_/O homogenization time was only 2 min. This could explain why the characteristics of the double emulsions prepared using a W_1_/O homogenization time of 2 min were intermediate between those obtained at 5 and 10 min. Homogenization times of 2 or 10 min both resulted in mean oil droplet size and dispersity below 500 nm and 0.25, respectively, and highly negatively charged droplets (below −40 mV). This suggests that decreasing PGPR-oil premixing speed from 10,000 rpm (for 2 min) to 5000 rpm (for 5 min) was compensated for when increasing W_1_/O homogenization time to 10 min.

The impact of W_1_/O homogenization time was greater on TA encapsulation efficiency than on TP’s, suggesting that anthocyanins may be more sensitive to the effect of homogenization time (first and second homogenization steps) and droplet characteristics than TP.

The wide range of encapsulation efficiencies reported in the literature [25,48,49,50] suggests that encapsulation efficiency depends not only on process parameters, but also on the nature of the compound(s) encapsulated. In the present work, anthocyanins may have been more prone to leaking out of the water and oil droplets due to their higher water solubility compared to other phenolics [48]. The characteristics of the primary W_1_/O emulsion and its adequate stabilization seem to be crucial to maximize the encapsulation efficiency of blueberry phenolics, anthocyanins in particular.

## 3. Materials and Methods

### 3.1. Plant Material and Chemicals

Blueberry pomace powder prepared by freeze-drying blueberry wine pomace from *Vaccinium augustifolium* was kindly provided by Nova Agri Inc. (Centreville, NS, Canada). The powder was stored at −30 °C prior to use. Food-grade corn oil was purchased from a local supermarket in Guelph, ON, Canada. Whey protein isolate (WPI) (97.5% *w*/*w* whey proteins, halal certified) was purchased from Davisco Foods International Inc. (Le Sueur, MN, USA) and polyglycerol polyricinoleate (PGPR 4145, hydrophilic to lipophilic balance 1.4) was donated by Palsgaard Inc. (Morris Plains, NJ, USA). All the chemicals were of analytical reagent grade. Ethanol, methanol and formic acid of HPLC grade were purchased from Caledon Laboratories (Georgetown, ON, Canada). Sodium benzoate and hydrochloric acid were obtained from Sigma-Aldrich (Oakville, ON, Canada). Sodium carbonate, Folin-Ciocalteu phenol reagent (2 N) and gallic acid were obtained from Sigma-Aldrich (St Louis, MO, USA). Anthocyanin standards (cyanidin chloride, delphinidin chloride, malvidin chloride, pelargonidin chloride, peonidin chloride and petunidin chloride) and dimethyl sulfoxide were purchased from Indofine Chemical Company Inc. (Somerville, NJ, USA).

### 3.2. Ultrasound-Assisted Extraction of Phenolic Compounds from Blueberry Pomace

Polyphenols were extracted by ultrasound-assisted extraction as described by Bamba et al. [32] using 50% ethanol as the solvent and a solid/solvent ratio of 1/20. Briefly, 2 g of blueberry pomace powder was transferred to a 125 mL amber-tinted flask, then 40 mL of 50% ethanol in milliQ water was added and gently mixed for a few minutes. The mixture pH was 6.3. Extraction was performed at 40 °C for 60 min in an ultrasonic cleaner bath (15.5 × 14 × 9 cm, Symphony 97043-932, VWR, Mississauga, ON, Canada) operated at maximum power (35 kHz, 64 W). The resulting extracts were centrifuged at 6000 rpm for 15 min at room temperature, followed by vacuum filtration through a 45 μm Millipore polyvinylidene difluoride (PVDF) membrane. The filtrate was transferred into a 100 mL amber glass volumetric flask wrapped with aluminum foil to prevent degradation of bioactive compounds and concentrated by rotary evaporation under vacuum (Büchi Rotavapor RII, Rose Scientific Ltd., Essen, Germany) at 40 °C and 100 mbar for 20 min. The liquid concentrated blueberry pomace extract (BPE) was stored at 4 °C in an amber-coloured bottle until further use. Its total phenolic content and total anthocyanin content were 35.95 ± 0.85 mg of gallic acid equivalents (GAE)/g and 91.93 ± 2.04 mg/g, respectively, on a dry matter basis, which corresponded to 3.64 ± 0.57 mg GAE/mL and 9.0 ± 1.02 mg/mL of liquid extract.

### 3.3. Preparation of the Double Emulsions

The W_1_/O/W_2_ double nanoemulsions were prepared by two-stage homogenization at ambient temperature using food-grade ingredients. The homogenization parameters that were varied are W_1_/O/W_2_ homogenization pressure, stirring speed and time, and W_1_/O homogenization stirring time, according to the experimental scheme summarized in Table 1. Water-in-oil (W_1_/O) emulsions were prepared during the first stage. The continuous oil phase consisted of 4 g of PGPR dispersed into 76 g corn oil at room temperature by stirring at 5000 rpm for 5 min, except where indicated, using a Polytron PT 2500E homogenizer (Kinematica AG, Luzern, Switzerland). Then, 20 g of BPE (W_1_) was carefully added drop by drop to the oil phase using a 3 mL disposable graduated transfer pipette, with continuous stirring at 5000 rpm for 5 min at room temperature. The final 20% (*w*/*w*) W_1_/O emulsions were made by homogenization using the Polytron at 10,000 rpm for a variable duration from 2 to 10 min. In the second stage, the primary W_1_/O emulsion (30 g) was slowly dispersed into a continuous secondary water phase (W_2_) consisting of 70 g of 2.5% *w*/*w* whey protein (WPI) solution (pH 6.8) and homogenized using the Polytron at variable stirring speed (3000 to 12,000 rpm) for 15 min, or at 6000 rpm for 10 to 20 min (Table 1). The WPI solution was prepared by dissolving 2.5 g of WPI and 0.02 g of sodium benzoate in 100 g of milliQ water, with continuous stirring at 500 rpm for 2 h at room temperature, followed by storage at 4 °C overnight to allow complete hydration of the proteins prior to use. The final W_1_/O/W_2_ emulsions (30% *w*/*w* W_1_/O and 70% *w*/*w* W_2_) were prepared by passing through a high-pressure homogenizer (Microfluidics M-110P Microfluidizer, ATS Scientific Inc., Burlington, ON, Canada) for one homogenization cycle at variable pressure from 50 to 200 MPa. The starting temperature for preparing all the emulsions was room temperature. All the experiments were conducted in triplicate. Measurements on the emulsions were made on fresh emulsions immediately after their preparation.

### 3.4. Total Phenolic and Anthocyanin Contents of Blueberry Pomace Extract and Emulsions

#### 3.4.1. Determination of Total Phenolic Content (TPC)

TPC was determined using the method of Folin-Ciocalteu described by Tournour et al. [57] with slight modifications [32]. Briefly, 25 μL of either sample (BPE or emulsion) or standard (gallic acid) properly diluted with milliQ water were transferred into appropriate wells. With a multichannel pipette, 125 μL of 0.2 N Folin-Ciocalteu reagent were added to each well, then the plate was swirled and incubated at room temperature in the dark. After 8 to 10 min, 125 μL of 7.5% sodium carbonate was added, then the solution was thoroughly mixed and incubated at room temperature for 30 to 60 min. Subsequently, the absorbance was recorded at 765 nm with a spectrophotometric microplate reader (Synergy HT Multi-Detection Microplate Reader, BioTek Instruments, Winooksi, VT, USA). Absorbance was compared to the gallic acid standard curve (R^2^ = 0.999) and TPC was expressed as mg of gallic acid equivalents per g of dry matter. Each standard and sample solution were analysed in triplicate.

#### 3.4.2. Determination of Total Anthocyanin Content (TAC)

TAC was determined by high-performance liquid chromatography with photodiode array detector (HPLC-PAD) using an Agilent 1100 series system (Agilent Technologies, Waldbronn, Germany) and a C-18 HPLC column (5 μm, 120 Å, 150 × 4.6 mm) (YMC Inc., Wilmington, NC, USA), as previously described [32] with some modification. The elution solvents were (A) 10% formic acid in milliQ water (*v*/*v*) and (B) 100% methanol. Solvent gradient was linear from 95% A/5% B to 40% A/60% B (0–20 min), isocratic at 40% A/60% B (20–23 min), linear from 40% A/60% B to 95%A/5% B (23–24 min), and isocratic at 95%A/5% B (24–28 min). Flow rate was 0.7 mL/min, column temperature 25 °C, pressure 300 bar, sample temperature was ambient and injection volume was 40 μL (BPE or emulsion). The detection wavelength was 520 nm. TAC was determined after acid hydrolysis with hydrochloric acid [28], which enables the determination of the aglycon forms of the anthocyanins, using a sample volume of 60 μL (BPE) or 2 mL (emulsion). After hydrolysis, the samples were filtered through a 0.25 μm polytetrafluoroethylene (PTFE) membrane filter into an HPLC vial and analysed by HPLC. Two replicates per sample were prepared. Commercially available anthocyanidin standards of cyanidin, delphinidin, malvidin and petunidin were separately dissolved in 2 mL dimethyl sulfoxide (99.9%) and used as standard stock solutions. New stock solutions were prepared each week to ensure freshness of the standards. For each standard, the stock solution was diluted in methanol to prepare 3.125, 6.25, 12.5, 25 and 50 μg/mL solutions used to generate the linear calibration curve (R^2^ > 0.997) of peak area against concentration. TAC was the sum of the four individual anthocyanins identified and quantified, namely cyanidin, delphinidin, malvidin and petunidin, expressed as mg per g of dry matter.

### 3.5. Characterization of the Double Emulsions

#### 3.5.1. Particle Size Analysis and Zeta Potential

Particle size, size dispersity (also known as polydispersity index, although dispersity is the term recommended by IUPAC [58]) and zeta potential of the oil droplets in the double emulsions were measured by dynamic light scattering using a Zetasizer Nano ZS90 (Malvern Instruments, Worcestershire, UK) with the optical detector positioned at a 90° angle. Three replicate measurements per sample were performed by the Zetasizer. For particle size, the surface-weighted mean diameter, d_3,2_, also known as Sauter diameter, was used (Equation (1)):

(1) $$d_{3,2}\;(\mathrm{nm})=\sum n_{i}d_{i}^{3}/\sum n_{i}d_{i}^{2}$$

where *n_i_* is the number of droplets of diameter *d_i_*. The zeta potential (in mV) is the electrical potential at the ‘shear plane’, which is defined as the distance away from the droplet surface below which the counter-ions remain strongly attached to the droplet when it moves in an electrical field [59]. The measurements were performed on fresh double emulsions immediately after their preparation. The double emulsions were diluted in milliQ water by a hundred times prior to measurement at room temperature.

#### 3.5.2. Encapsulation Efficiency

Encapsulation efficiency was determined as the percentage of total phenolics (TP) and total anthocyanins (TA) trapped by encapsulation within the inner aqueous phase (W_1_) following W_1_/O/W_2_ emulsification, as described by Mohammadi et al. [60] with slight modifications. A volume of 4 mL of double emulsion was transferred into a 16 mL centrifuge tube, diluted two times by adding 4 mL of milliQ water, then centrifuged (Labnet Hermle Z206A Centrifuge, Mandel, Guelph, ON, Canada) at 6000 rpm at room temperature for 45 min. The lower aqueous phase containing the non-encapsulated phenolic compounds was carefully collected using a syringe and analysed for TPC and TAC, using sample volumes of 25 μL for TPC analysis and 2 mL for TAC analysis. Encapsulation efficiency (%) was calculated using Equation (2):

Encapsulation efficiency (%) = 100 − (A_2_ × 100/A_1_) (2)

where A_2_ is the amount of TP or TA (in mg) in the lower aqueous phase containing the non-encapsulated phenolics, and A_1_ is the amount of TP or TA that was used to prepare the emulsion (21.84 mg GAE and 54.10 mg, respectively).

### 3.6. Statistical Analyses

Descriptive statistics were calculated and expressed as means ± standard deviation (SD). After checking for normality, means were compared using one-way analysis of variance (ANOVA) followed by Tukey’s multiple comparison test, or the Kruskal-Wallis test followed by U Mann-Whitney multiple comparison test, as appropriate. For the data expressed as percentage or fraction, the data were transformed using the log_10_ transformation prior to analysis as recommended [61]. Statistical analyses were performed using Statistica version 7 (StatSoft, Tibco Software, Paris, France) with statistical significance established at *p* ≤ 0.05.

## 4. Conclusions

A food-grade double nanoemulsion system was successfully developed to coencapsulate the total phenolics and anthocyanins extracted from blueberry pomace. The aqueous polyphenolic-rich extract (W_1_) was emulsified in corn oil with PGPR, while the oil droplets of the W_1_/O/W_2_ double emulsion were stabilized by a macromolecular emulsifier (WPI). Encapsulation efficiency and the size and charge characteristics of the emulsion droplets were significantly affected by homogenization pressure, stirring speed and time. High encapsulation efficiencies of TP and TA above 80% were achieved under homogenization conditions that resulted in well-controlled double emulsions by producing small oil droplets (average diameter below 400 nm) with relatively low size dispersity (below 0.25) and a high negative surface charge (−40 mV or less). This shows that nanoscale double emulsions are a promising method for coencapsulating total phenolics and anthocyanins from blueberry. Superior encapsulation efficiency of TP and TA was obtained by homogenizing for 10 min at 10,000 rpm (primary W_1_/O emulsion), followed by 15 min at 6000 rpm and microfluidization at 50 MPa (W_1_/O/W_2_). Stirring time had a greater impact on TA encapsulation efficiency than on TP’s and TA encapsulation yield was generally lower. This finding deserves consideration when high levels of anthocyanins or specific proportions of TA relative to TP are desired in the nanoparticulate carrier. These findings are helpful for developing nanocarriers systems suitable for the different phenolic fractions found in blueberry pomace for their incorporation in functional foods and other products with health claims. Future research is warranted to optimize process parameters and to assess the distribution of TP and TA between the different phases, their bioactivity upon encapsulation and their release characteristics.

## Figures and Tables

**Table 1:** Experimental conditions for the coencapsulation of polyphenols and anthocyanins from blueberry pomace by double emulsion produced by two-stage homogenization.

	Stage 2			Stage 1
	Final Double Emulsion	Coarse Double Emulsion		Primary Emulsion
	W_1_/O/W_2_ Homogenization (Microfluidizer) Pressure (MPa)	W_1_/O/W_2_ Homogenization (Polytron) Stirring Speed (rpm)	W_1_/O/W_2_ Homogenization (Polytron) Stirring Time (min)	W_1_/O Homogenization (Polytron) Stirring Time (min) *
Runs 1–3 (Figure 1)	50–100–200	6000	15	10
Runs 4–6 (Figure 2)	50	3000–6000–12,000	15	10
Runs 7–9 (Figure 3)	50	6000	10–15–20	10
Runs 10–12 (Figure 4)	50	6000	15	2–5–10

Emulsion composition: W_1_/O: 76 g corn oil, 4 g PGPR, 20 g of aqueous blueberry pomace extract (W_1_); W_1_/O/W_2_: 30 g W_1_/O in 70 g W_2_ (2.5% *w*/*w* whey protein isolate (WPI) solution, pH 6.8). * W_1_/O homogenization stirring speed: 10,000 rpm in all experiments. Experiments were conducted in triplicate.
